# The GEOS/ECCO C1440-LLC2160 Coupled Atmosphere-Ocean Simulation Dataset

**DOI:** 10.1038/s41597-026-07349-2

**Published:** 2026-05-12

**Authors:** Dimitris Menemenlis, Andrea Molod, Christopher N. Hill, Atanas Trayanov, Ehud Strobach, Jean-Michel Campin, Abdullah A. Fahad, Patrick Heimbach, Hong Zhang, Young-Kwon Lim, Sina Khani, Christopher E. Henze, David A. Ellsworth, Nina McCurdy

**Affiliations:** 1 Moss Landing Marine Laboratories, San José State University, Moss Landing, CA USA; 2 NASA, Goddard Space Flight Center, Greenbelt, MD USA; 3 Massachusetts Institute of Technology, Cambridge, MA USA; 4 Science Systems and Applications Inc., Lanham, MD USA; 5 Volcani Agricultural Research Organization, Rishon Letzion, Israel; 6 ESSIC, University of Maryland, College Park, MD USA; 7 Jackson School of Geosciences, University of Texas at Austin, Austin, TX USA; 8 Jet Propulsion Laboratory, CAlifornia Institute of Technology, Pasadena, CA USA; 9 University of Maryland Baltimore Campus, Baltimore, MD USA; 10 SSAI, Science Systems and Applications, Inc., Lanham, MD USA; 11 NASA Ames Research Center, Mountain View, CA USA

**Keywords:** Ocean sciences, Climate sciences

## Abstract

NASA’s Goddard Earth Observing System (GEOS) infrastructure was used to couple a cloud-admitting (7-km grid, 72 levels) configuration of the GEOS atmospheric model with a mesoscale-resolving (2–4-km grid, 90 levels) Estimating the Circulation and Climate of the Ocean (ECCO) configuration of the Massachusetts Institute of Technology general circulation model (MITgcm), and to conduct a 14-month “nature” simulation initialized with January 20, 2020, 21Z conditions. The output of this simulation is contained in the dataset described here. The NASA GEOS/ECCO Coupled Nature Run includes astronomical tidal forcing in the ocean component of the simulation, an interactive aerosol component and aerosol-cloud interactions in the atmosphere, and the storage of copious amounts of model output. The inclusion of tidal forcing permits a more realistic representation of vertical mixing in the ocean and of high-frequency variability that is aliased in satellite observations. All of the above make this simulation well suited as a nature run in Observing System Simulation Experiments (OSSEs) and for the study of high frequency/wavenumber coupled processes in weather and climate.

## Background & Summary

We describe a first-of-its-kind coupled Earth system model *Nature Run* dataset. The term “nature run” refers to a synthetic but known “truth” that can be used to study physical processes and to evaluate data assimilation methodologies and notional observing platforms before formulation, design, and deployment in the context of an Observing System Simulation Experiment (OSSE). The simulation used to produce the dataset was carried out using NASA’s Goddard Earth Observing System (GEOS) Atmospheric General Circulation Model (AGCM) and infrastructure developed by NASA’s Global Modeling and Assimilation Office (GMAO) coupled to the Massachusetts Institute of Technology general circulation model (MITgcm), which is at the heart of the Estimating the Circulation and Climate of the Ocean (ECCO) project. This “GEOS/ECCO” simulation is unique because of the model’s horizontal resolution, the 14-month-long time span, the inclusion of aerosol-cloud interactions in the atmospheric component, the inclusion of tidal forcing in the oceanic component, and the frequency and scope of the model output fields that have been saved.

The GEOS/ECCO simulation is based on two, separately-developed components that were coupled together for many different kinds of experiments. The first component is the GEOS AGCM, which has been used to conduct global atmospheric simulations with prescribed ocean conditions at increasingly higher resolutions, including a short-period integration with horizontal grid spacing as small as 1 km as part of the NOAA Hazardous Weather Testbed (HWRT)^1^. The high-resolution GEOS AGCM simulations have been used to participate in the model intercomparison on the dynamics of the atmospheric general circulation modeled on non-hydrostatic domains (DYAMOND)^2^, to examine the resolution dependence of simulated precipitation^3^, to evaluate the atmospheric energy spectra^4^, and for the simulation of tropical cyclones^5,6^. GEOS AGCM simulations at varying resolutions have also been used to evaluate the behavior of atmospheric convective parameterizations through the gray zone^7,8^ and the results of the sub-5 km-resolution simulations have been used in the development of subgrid-scale parameterizations for use in coarser-resolution simulations^9,10^.

The second component, developed by the ECCO project, has been used to carry out global-ocean simulations at increasingly-higher resolutions: 1/6^°^ nominal horizontal grid spacing in 2005^11^, 1/16^°^ grid spacing in 2007^12^, culminating with 1/24^°^ and 1/48^°^ grid spacing in 2016^13^. A key characteristic of this last set of ECCO ocean simulations is the inclusion of tidal forcing, which enables the generation of internal tides as well as of an increasingly more realistic internal gravity wave spectrum as model resolution is increased^14^.This last set of ECCO high-resolution simulations have led to an explosion of new scientific and practical applications, 204 refereed publications (at last count) on topics that include studies of air-sea interactions^15^, surface gravity waves^16^, internal waves^17–21^, ocean mesoscale and submesoscale variability^13,22–25^, vertical transports^26–28^, sea-ice^29^, bathymetry-steered currents^30,31^, biological dispersion^32–36^, machine learning^37^, and satellite mission design^38–43^.

The many publications based on high-resolution GEOS and ECCO nature runs are a testament to the utility of such simulations for addressing scientific and practical questions. A key limitation, however, is the prescribed ocean conditions used in GEOS simulations and the prescribed atmospheric conditions used in ECCO simulations. In order to more realistically represent air-sea interactions, a number of modeling groups are carrying out coupled, high-resolution, ocean-atmosphere simulations^44–46^. Phase II of the DYnamics of the Atmospheric general circulation Modelled On Non-hydrostatic Domains (DYAMOND) intercomparison project^47^ calls for the optional submission of sub-5km atmosphere-ocean-cryosphere coupled simulations, and only NASA and the ICOsahedral Nonhydrostatic (ICON) model developed at the Max Planck Institute for Meteorology^48^ submitted such simulation results. The first 40 days of the GEOS/ECCO Nature Run described herein is NASA’s coupled ocean-atmosphere model contribution to the DYAMOND Phase II project.

The GEOS/ECCO Nature Run was subsequently extended to 14 months of simulation, which makes it well-suited for Observing System Simulation Experiment (OSSE) applications, such as the development of a winds-and-current satellite mission^43^. The GEOS/ECCO simulation is also being evaluated for use in OSSEs by NOAA’s Quantitative Observing System Assessment Program (QOSAP) and by the UN Ocean Decade Project’s Synergistic Observing Network for Ocean Prediction (SynObs). In addition to OSSE applications, the GEOS/ECCO simulation provides a virtual laboratory for exploring coupling across scales in the atmosphere and ocean and for the study of high frequency/wavenumber processes in weather and climate. The high spatiotemporal frequency of the model output archive enables the study of a richer set of atmosphere, ocean, and air-sea exchange dynamics than was previously possible^49^, and the inclusion of tidal forcing permits a more realistic representation of high-frequency variability and vertical mixing processes in the ocean^21,50,51^. A few early studies based on the GEOS/ECCO Nature Run are beginning to be published^3,43,52–54^.

## Methods

The main components of the GEOS/ECCO model are the GEOS Atmospheric General Circulation Model (AGCM)^55,56^, the Goddard Chemistry Aerosol Radiation and Transport (GOCART) model^57,58^, a catchment-based land surface model^59^, the CICE 4.1 sea ice thermodynamics model^60^, an atmosphere-ocean skin layer^61^, and the ocean and sea-ice dynamics components of the MITgcm^62–64^. The above model components, which are coupled by leveraging the GEOS coupled model infrastructure, are described below. The coupled DYAMOND simulation presented here, in keeping with the DYAMOND Phase II protocol, is a free-running simulation with no nudging, adjustments, flux corrections, or data assimilation.

### GEOS Atmosphere, Aerosol, Land, and Ice Components

The GEOS AGCM dynamical core simulates large-scale transport and dynamics with an adaptation of the Flux-Form Semi-Lagrangian (FFSL) Finite-Volume (FV) dynamics^65^ for a cubed sphere horizontal discretization^66^. The GEOS AGCM includes a suite of physical parameterizations for moist processes, radiation, turbulence, and gravity wave drag. Convection is parameterized using the Relaxed Arakawa-Schubert scheme^67^, prognostic cloud cover and cloud water and ice are determined by a two-moment cloud microphysics parameterization^68^. A specified Probability Distribution Function (PDF) for total water governs the cloud macrophysics^10^. Longwave^69^, and shortwave^70,71^ radiative transfer are parameterized as band models. The turbulence parameterization is a combination of the Lock scheme^72^ and a Richardson-number-based algorithm^73^, and the surface layer turbulence is a Monin-Obukhov parameterization^74^ with an ocean surface roughness that is determined by a polynomial approximation to a combination of algorithms valid in different wind regimes^75–78^. The gravity wave drag parameterization computes momentum and heat deposition due to orographic^79^ and nonorographic^80^ waves.

The GEOS AGCM is coupled to the GOCART^58,81^ aerosol model that predicts dust, sea salt, sulfate, nitrate, organic carbon and black carbon, each in several size bins. The prediction of aerosol is critical for the prediction of aerosol-cloud interaction in the two-moment cloud microphysics, and therefore has a profound impact on the model’s clouds and cloud forcing. GOCART’s anthropogenic emissions and biomass burning are prescribed, and emissions of dust and sea salt are wind driven^82,83^. Sea salt emission is also modulated with a Sea Surface Temperature (SST)-derived correction^84^. Biomass burning emissions have daily variability and are from the Quick Fire Emissions Dataset (QFED) version 2.4-r6^85^. GOCART models the transfer of aerosol among the size bins, and aerosols in each bin are transported by advection and during convection. Aerosols also undergo scavenging and dry deposition.

The GEOS AGCM is also coupled to a Land Surface Model^59^, which is a catchment-based scheme that treats subgrid scale heterogeneity in surface moisture and heat statistically. The glacier model is simple, in which glacial thermodynamic processes are parameterized using an adaptation of a snow model^86^ to simulate glacial ice^87^, and the catchment and glacier models are each coupled to a multi-layer snow model^86^.

The configuration of the GEOS AGCM, aerosol, and land models used for the GEOS/ECCO Nature Run simulation is the ‘c1440’ horizontal grid, a cubed sphere grid with approximately 7-km horizontal grid spacing, and a hybrid *η*-pressure vertical coordinate with 72 levels, approximately eight of them in the atmospheric boundary layer and with a lid at 0.01 hPa. Over the open ocean, a skin layer parameterization^61^ is configured to impose a maximum delay of one-hour on the interaction between the ocean and the atmosphere. The CICE 4.1 sea ice model^60^ is used for thermodynamic computations only, while the MITgcm, described next, includes the sea ice dynamical transport and ridging.

### High-resolution ECCO configuration of the MITgcm

**Fig. 1:**
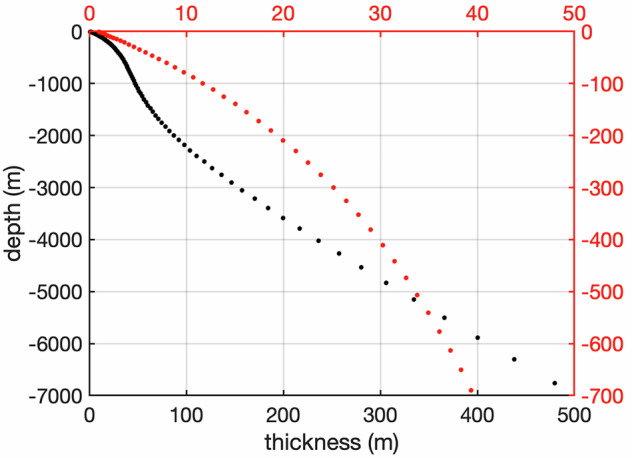
Ocean model thicknesses as a function of depth.

For the GEOS/ECCO Nature Run, the MITgcm^62,63^ has been configured to solve the incompressible hydrostatic Boussinesq equations for fluid motion in a rotating frame of reference using a nonlinear free-surface and real freshwater flux^88^, and the K-Profile Parameterization (KPP) scheme^89^ for vertical mixing. The MITgcm horizontal grid is the so-called “Lat-Lon-Cap” grid^90^, and was configured here to run at LLC2160, which has a nominal grid spacing of 2–4 km. The vertical discretization is in rescaled height coordinates^88^, with 90 vertical levels ranging in thickness from 1 m near the surface to 480 m at a maximum depth of 7 km. The locations of the levels and the thickness of each level are shown in Fig. 1.

### Description of the Simulation

**Fig. 2:**
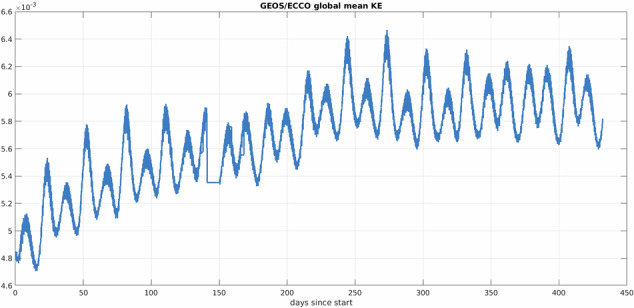
Time series of the global mean total ocean column kinetic energy in *kg* *m*^2^ *s*^−2^.

Initial conditions for the atmosphere, land and aerosol components of the simulation were obtained from fields at the ‘c1440’ resolution that were part of the simulations performed by GMAO to initialize the atmosphere-only DYAMOND simulation (https://gmao.gsfc.nasa.gov/gmao-products/dyamond-phase-ii/data-access_dyamond-phase-ii/). Initial conditions for the predicted cloud fields were zeroes, allowing the clouds to be bootstrapped. The initial conditionsfor the ocean were obtained from ECCO experiments at the ‘llc2160’ resolution. A set of spinup experiments were initialized withthese fields that cycled through the first month several times, primarily to allow thesea ice fields from the atmosphere and ocean to adjust. After 3 such cycles the coupled DYAMOND simulation was begun. The spinup period for the ocean at depth was determined by examining the time series of the ocean column total global mean kinetic energy (Fig. 2). Based on a ‘flattening’ of the kinetic energy at approximately 200 days into the simulation, the results shown here will all allow for a 200 day spinup period.

In addition to the initial conditions, boundary conditions of two different types were used as part of the simulation. The first set of boundary conditions are related to the state of the earth’s surface, related to surface type, topography, and vegetation^91^, and the second are the emissions of aerosol components^81^.

The simulation’s internal timestep (“heartbeat”) was 45 seconds, and the ocean/atmosphere coupling, the atmospheric physics, dynamics, and land model all used that timestep. The atmospheric radiation used a 900 second timestep. Runoff from the land (excess of precipitation minus evaporation minus moisture absorbed into the soil) was used to calculate discharge into the ocean. The USGS topological dataset was used to define the river basins using the Pfafstetter coding^92^. Pairs of river mouth destination points and land source points are defined, and runoff from the land is accumulated and instantaneously converted to discharge.

Details of the atmospheric and the oceanic configuration of the GEOS/ECCO model used for the simulation presented here can be found at: https://github.com/geos-mitgcm-ecco/gmao_mitgcm_coupling/tree/master/DYAMOND/experiments/c1440_llc2160_02 in the code, input and geos subdirectories.

The simulation ran on 8160 Xeon Haswell processor cores of the Pleiades Supercomputer at the NASA Ames Research Center. The operating system was SUSE Linux Enterprise Server 11 (x86_64) and used Linux kernel 3.0.We used the Message Passing Interface (MPI) from a custom version of SGI’s Message Passing Toolkit (MPT) version 2.10.The numerical code was compiled using Intel Fortran and C Compilers, version 12.1.4. The model throughput was 3 simulated days per wallclock day. Due in part to the data needs of a nature run, and to the protocol for output in the DYAMOND intercomparison, extensive output fields were generated, which include hourly 3-dimensional fields from the atmosphere and the ocean, and 15-minute frequency output of a select number of 2-dimensional fields from the atmosphere and the ocean. The total output of the 14-month simulation is approximately 2.5 Pb in size. To achieve this amount of output while maintaining a tractable simulation time, input/output was configured to run asynchronously.

## Data Records

The dataset described here is available at https://www.poseidon-ocean.net/products/datasets/^93^, and instructions for downloading the data can be found at: https://www.poseidon-ocean.net/access-process-for-the-dyamond-dataset-on-sciserver/. The dataset contains 27 GEOS output ‘collections’ and 24 MITgcm file types, totaling approximately 2.5 Pb in volume. Animations and still images of atmospheric and oceanic fields are available at https://data.nas.nasa.gov/geoseccoviz. In all, 357 GEOS fields and 31 MITgcm fields have been visualized. For each field, there are two map projections: latitude/longitude and North and South polar projections. Additionally, 21 MITgcm fields are visualized with polar projection to ±50^°^ latitude, which permits a more detailed view of atmosphere-ocean interactions with sea ice. For each visualization there are three resolutions: HD (1920 × 1080) animation, 4K (3840 × 2160) animation, and 4K still image. In all, 2391 visualizations have been created and are available to facilitate a first look of the simulated fields.

The GEOS output is organized into a set of ‘collections’ that share a common base name and a prescription for templating the dates. The GMAO file name convention also calls for character strings or nodes that indicate whether the fields are averaged in time or instantaneous, the frequency of output, whether 2 dimensional or 3 dimensional, a descriptor of the content, and a descriptor of the output grid. All GEOS output fields have the grid descriptor ‘Mv’ or ‘Mx’, ‘Mv’ to indicate that fields are output on the native cubed sphere grid and on the model’s native ‘eta’ levels, and ‘Mx’ to signify the native horizontal grid and a 2-dimensional field. The list of fields required by the DYAMOND protocol is augmented with additional output. The collections and a brief description of the fields contained within are presented here:

- geosgcm_surf: Hourly averages of Various 2-dimensional prognostic and diagnostic fields and vertically integrated 3-dimensional diagnostic fields. Fields include vertically integrated cloud fractions and optical depths, components of the precipitation, turbulent exchange coefficients, surface albedos, surface soil moistures and temperatures, 2-meter and 10-meter temperatures.
- geosgcm_turb: Daily averages of Atmospheric 3-dimensional turbulence exchange coefficients and Richardson Number.
- geosgcm_tend: Daily averages of the 3-dimensional different ‘tendency’ or budget terms that constitute the changes to the atmospheric prognostic fields.
- tavg_01hr_3d_WU_Mv: One hour time averages of the 3-dimensional product of the atmospheric vertical velocity and atmospheric zonal velocity.
- tavg_01hr_3d_WV_Mv: One hour time averages of the 3-dimensional product of the atmospheric vertical velocity and atmospheric meridional velocity.
- tavg_01hr_3d_U_Mv: One hour time averages of the 3-dimensional atmospheric zonal velocity.
- tavg_01hr_3d_V_Mv: One hour time averages of the 3-dimensional atmospheric meridional velocity.
- tavg_01hr_3d_W_Mv: One hour time averages of the 3-dimensional atmospheric vertical velocity.
- tavg_01hr_3d_H_Mv: One hour time averages of the 3-dimensional atmospheric geopotential height.
- tavg_01hr_3d_CNVMFD_Mv: One hour time averages of the 3-dimensional detrained convective mass flux.
- tavg_01hr_3d_CNVMFC_Mv: One hour time averages of the 3-dimensional total convective mass flux.
- tavg_15mn_2d_flx_Mx: 15-minute averages of various 2-dimensional fields including precipitation and surface turbulent and radiative fluxes of mass, momentum and energy.
- const_2d_asm_Mx: Constant 2-dimensional describing the surface type (Land, Lake, Glacier, Ocean), the surface elevation and the area of model grid boxes.
- inst_01hr_3d_DTHDT_Mv: Instantaneous 3-dimensional tendency of atmospheric potential temperature due to moist processes, sampled hourly
- inst_01hr_3d_DTHDTCN_Mv: Instantaneous 3-dimensional tendency of atmospheric potential temperature due to convection, sampled hourly.
- inst_01hr_3d_CO_Mv: Instantaneous 3-dimensional atmospheric Carbon Monoxide, sampled hourly.
- inst_01hr_3d_CO2_Mv: Instantaneous 3-dimensional atmospheric Carbon Dioxide, sampled hourly.
- inst_01hr_3d_DELP_Mv: Instantaneous 3-dimensional Pressure thickness of each atmospheric vertical layer, sampled hourly.
- inst_01hr_3d_P_Mv: Instantaneous 3-dimensional atmospheric Pressure, sampled hourly.
- inst_01hr_3d_T_Mv: Instantaneous 3-dimensional atmospheric Temperature, sampled hourly.
- inst_01hr_3d_T_Mv: Instantaneous 3-dimensional atmospheric Temperature, sampled hourly.
- inst_01hr_3d_RL_Mv: Instantaneous 3-dimensional atmospheric Liquid Cloud Drop Radius, sampled hourly.
- inst_01hr_3d_RI_Mv: Instantaneous 3-dimensional atmospheric Ice Cloud crystal size, sampled hourly.
- inst_01hr_3d_FCLD_Mv: Instantaneous 3-dimensional Cloud Fraction, sampled hourly.
- inst_15mn_2d_prs_Mx: Instantaneous Atmospheric Vertical pressure velocity, relative humidity and geopotential height at 850, 700, 500 and 200 hPa, sampled every 15 minutes.
- inst_15mn_2d_asm_Mx: Instantaneous 2-dimensional fields of surface pressure, 10-meter winds, surface and 2-meter temperature and humidity, and vertically-integrated cloud water and cloud ice, Cumulus Available Potential Energy (CAPE), and convective inhibition, sampled every 15 minutes.

The MITgcm output is organized into files that contain one output field per time step each, with a file name that contains a node with the field name, one with the model time step of output, followed by the node ‘.data’. The model time step can be converted to a date and time by multiplying by 45 s and adding the starting time (January 19, 2020, 21:00:00). The simulation ends on MITgcm time step 829200 (March 26, 2021, 18:00:00). The MITgcm fields saved during the simulation are:

- Eta: 2D Surface height anomaly (m). In the presence of sea ice, the area density of the ice and snow press down on Eta.
- KPPhbl: 2D KPP boundary layer depth (m).
- PhiBot: 2D Bottom pressure potential anomaly (m^2^/s^2^).
- SIarea: 2D Fractional ice-covered area for 5 categories (0 to 1).
- SIheff: 2D Effective ice thickness for 5 categories (m).
- SIhsnow: 2D Effective snow thickness for 5 categories (m).
- SItice: 2D Ice surface temperature for 5 categories (deg K).
- SIuice: 2D Zonal (relative to grid) ice velocity, positive from West to East (m/s).
- SIvice: 2D Meridional (relative to grid) ice velocity, positive from South to North (m/s).
- Salt: 3D Salinity (g/kg).
- Theta: 3D Potential temperature (deg C).
- U: 3D Zonal (relative to grid) velocity, positive from West to East (m/s).
- V: 3D Meridional (relative to grid) velocity, positive from South to North (m/s).
- W: 3D Vertical velocity (m/s).
- oceFWflx: 2D Net upward freshwater flux, positive increases salinity (kg/m^2^/s).
- oceQnet: 2D Net upward surface heat flux (including shortwave), positive decreases theta (W/m^2^).
- oceQsw: 2D Net upward shortwave radiation, positive decreases theta (W/m^2^).
- oceSflux: 2D Net upward salt flux, positive decreases salinity (g/m^2^/s).
- oceTAUX: 2D Zonal (relative to grid) surface wind stress, positive increases uVel (N/m^2^).
- oceTAUY: 2D Meridional (relative to grid) surface wind stress, positive increases vVel (N/m^2^).

Note that the MITgcm unit of salinity is g/kg, which is approximately equivalent to a Practical Salinity Unit (PSU). Also note that U, V, oceTAUX, oceTAUY, SIuice, and SIvice are aligned relative to the model grid, not geographical coordinates, and that they are specified at the SouthWest C-grid velocity points.The vertical velocity W is specified at the top of the grid cell. All other scalar fields are specified at the tracer point, i.e., the center of each grid cell. In the presence of sea ice, oceFWflx, oceQnet, oceQsw, oceSflux, oceTAUX, and oceTAUY include the ice-to-ocean components of the fluxes, in addition to the fractional atmosphere-to-open-ocean component, that is, the net flux acting on the “wet” ocean. Subdirectory grid contains a description of the MITgcm grid. In addition to above fields, that were saved during the simulation, we also provide the following derived or computed fields:

- PS: Ocean surface atmospheric pressure at grid cell center (Pa).
- U10M: 10-meter Eastward Wind Velocity at grid cell center (m/s).
- V10M: 10-meter Northward Wind Velocity at grid cell center (m/s).
- SSH: Sea Surface Height at grid cell center (m).
- SSH_notides: SSH with tides removed at grid cell center (m).
- SSH_noIB: SSH_notides with Inverse Barometer (IB) correction at grid cell center (m).
- SSH_steric: Steric component of SSH at grid cell center (m).

Fields PS, U10M, and V10M are derived from the GEOS inst_15mn_2d_ams_Mx diagnostics and interpolated to the MITgcm grid. Contrary to oceTAUX and oceTAUY, which are specified at the SouthWest C-grid velocity points and aligned relative to the MITgcm grid, U10M and V10M are specified at the center of the grid cell and aligned with geographic Eastward and Northward directions. Contrary to Eta, the mass of the snow and ice are included in SSH, that is:

1 $$\,{\rm{SSH}}={\rm{Eta}}+({\rm{SIheff}}\,\ast {\rm{SEAICE}}\_{\rm{rhoIce}}+\,{\rm{SSIhsnow}}\,\ast {\rm{SEAICE}}\_{\rm{rhoSnow}})/{\rm{rhoConst}}\,,$$

where SEAICE_rhoIce = 910 kg/m^3^, SEAICE_rhoSnow = 330 kg/m^3^, and rhoConst = 1027.5 kg/m^3^. Tides are removed in SSH_notides using T_TIDE^94^. The inverse barometer correction is computed as follows:

2 $${\rm{SSH}}\_{\rm{noIB}}={\rm{SSH}}\_{\rm{notides}}+({\rm{PS}}-{\rm{GlobalMean(PS)}})/{\rm{rhoConst}}/g,$$

where *g* = 9.81 m/s^2^ is the acceleration due to gravity. The steric component of SSH is approximated^40,95^:

3 $${\rm{SSH}}\_{\rm{steric}}=\,{\rm{SSH}}-({\rm{PhiBot}}-{\rm{TimeMean}}({\rm{PhiBot}})+({\rm{PS}}-{\rm{GlobalMean(PS)}})/{\rm{rhoConst}})/g,$$

where TimeMean(PhiBot) is evaluated during the last 12 months of the GEOS/ECCO Nature Run.

The vertical distribution of shortwave radiation in the upper ocean is not provided in the output fields, but may be computed by using the code available in the MITgcm code base. The calculation is done in two steps. First, https://github.com/MITgcm/MITgcm/blob/decd05a68a16c9a10b05da054f140886b217a441/model/src/apply_forcing.F#L680 applies SW radiation to heat tendency. This code is used to compute the hourly output net shortwave radiation(called “oceQsw”). The second step uses: https://github.com/MITgcm/MITgcm/blob/decd05a68a16c9a10b05da054f140886b217a441/model/src/ini_forcing.F#L181 and the routine https://github.com/MITgcm/MITgcm/blob/master/model/src/swfrac.F to compute the three-dimensional profile of shortwave radiation reaching the ocean.

## Technical Evaluation

The use of a nature run as part of an OSSE or for modeling studies or parameterization development hinges on the scientific fidelity of the simulation. This fidelity includes the time-mean behavior and variability on the time scales relevant for predictability at different lead times. Here we present an evaluation of the atmospheric and oceanic fields.

### Atmosphere

For technical validation of the atmosphere we present comparisons of seasonal mean wind and temperature fields to demonstrate the fidelity of the average properties of the simulation. This can be regarded as the first order evaluation. The evaluation of these mean fields entails a comparison with results from the Modern-Era Retrospective Analysis for Research and Applications, Version 2 (MERRA-2)^96^. Given the free-running nature of the simulation to be evaluated here, and the lack of predictability beyond synoptic scales^97^, evaluation of the atmospheric is conducted by comparing against 2-year averaged fields from MERRA-2, from 2000-2020. Variations on subseasonal time scales (on the order of 30-60 days) rely largely on the fidelity of the simulation of the Madden-Julian Oscillation (MJO), and the MJO will be evaluated here as it transitions through its different phases. Of particular interest to the OSSE community is the evaluation of the extrema associated with tropical cyclones, and track counts at various tropical cyclone strengths will be evaluated here.

#### Time-Mean Fields

**Fig. 3:**
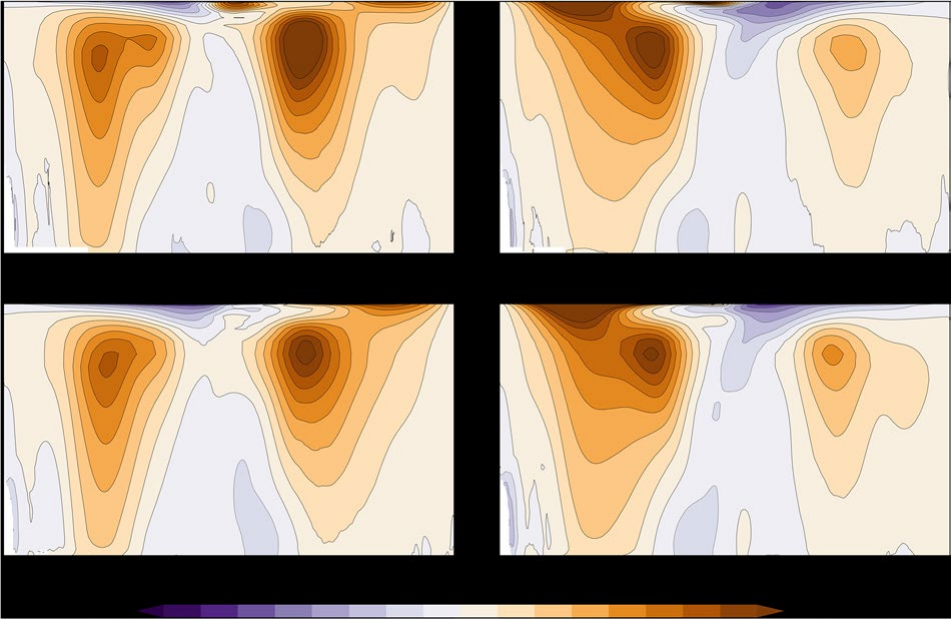
Seasonal mean zonal mean of the zonal component of the wind in *msec*^−1^. (**a**) December-January-February (DJF) average from the GEOS/ECCO Nature Run, (**b**) Same as (**a**) but for the June-July-August (JJA) seasonal mean, (**c**) Same as (**a**) but the MERRA-2 field is shown, (**d**) Same as (**b**) but the MERRA-2 field is shown.

**Fig. 4:**
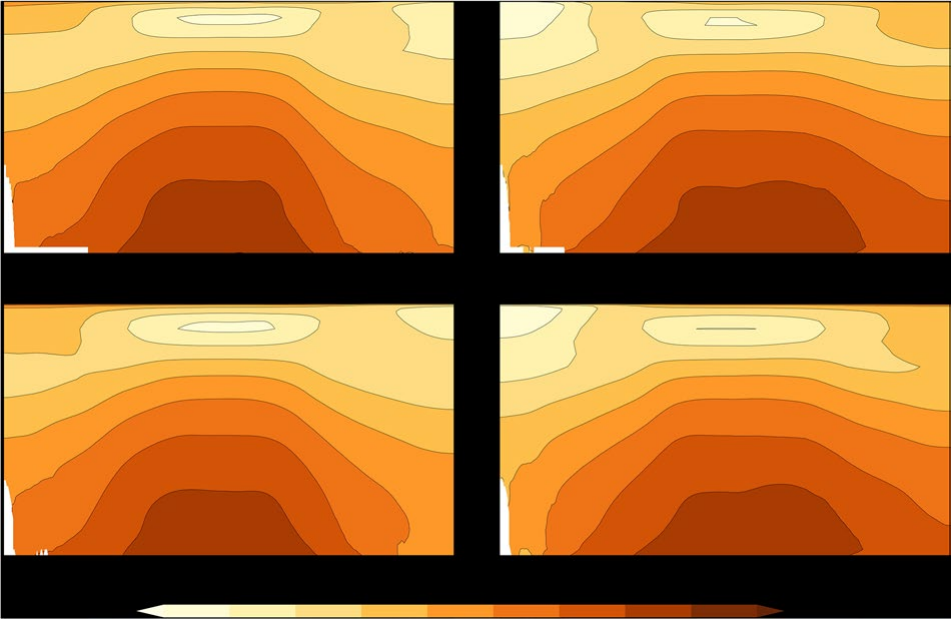
Seasonal mean zonal mean of the atmospheric temperature in ^°^*K*. (**a**) December-January-February (DJF) average from the GEOS/ECCO Nature Run, (**b**) Same as (**a**) but for the June-July-August (JJA) seasonal mean, (**c**) Same as (**a**) but the MERRA-2 field is shown, (**d**) Same as (**b**) but the MERRA-2 field is shown.

The zonal mean zonal wind fields for each season of the simulation are shown in Fig. 3. The pattern of the simulated fields matches well with MERRA-2 fields in both seasons, and the errors in the simulation are comparable with state of the art coupled model errors^98^. The subtropical jet is stronger in the nature run by up to 2 ${\rm{m}}\,{\sec }^{-1}$ in the winter hemisphere in both seasons, and weaker in the summer hemisphere in JJA by up to 2 ${\rm{m}}\,{\sec }^{-1}$ in the summer hemisphere. The stratospheric easterlies are stronger in the nature run simulation than in MERRA-2 in the JJA summer hemisphere by up to 2 ${\rm{m}}\,{\sec }^{-1}$. The zonal mean temperature fields for DJF and JJA are shown in Fig. 4. In general, the temperatures are within 1K of MERRA-2 temperatures. The differences in the temperature and wind fields from MERRA-2 are well within the expected differences between a simulation and a reanalysis^99^.

#### Variability

**Fig. 5:**
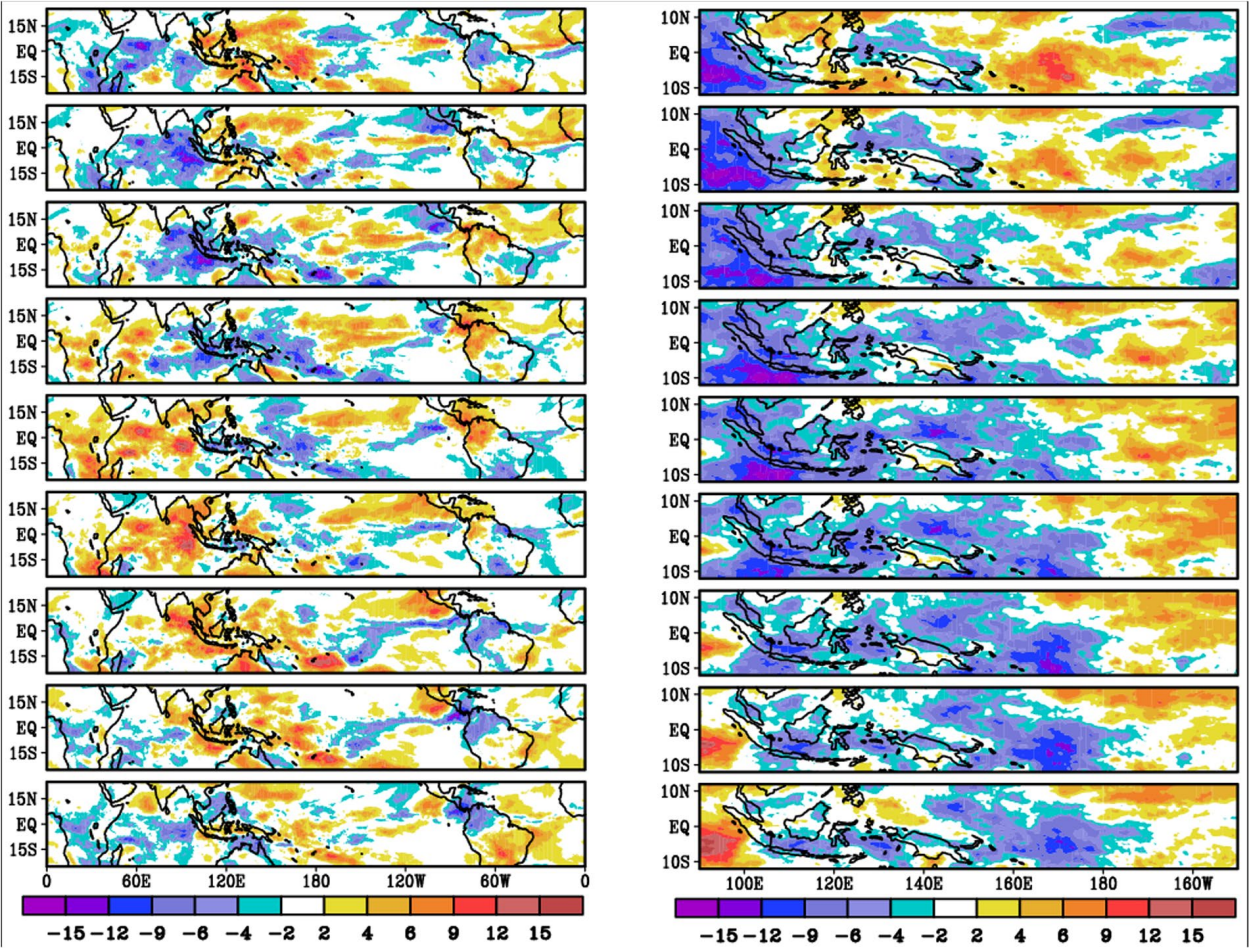
Left hand panel shows the tropical Outgoing Longwave Radiation (OLR) zonal anomalies (20-60 day low pass filtered) in 5-day increments as the Madden Julian Oscillation (MJO) propagates eastward from top to bottom. The right hand panel shows a sequence of low pass filtered OLR in 2-day increments as the MJO traverses the Maritime Continent.

The Madden-Julian oscillation (MJO) is the dominant mode of tropical intraseasonal variability and provides a major source of tropical and extratropical predictability on subseasonal time scales (eg.^100^). The MJO is an eastward moving tropical disturbance of clouds, rainfall, winds, and pressure that circles the tropics in 30 to 60 days, on average. The MJO is also connected dynamically with many other modes of atmospheric and coupled tropical variability, and the teleconnections from the MJO also show connections to mid-latitude variability from weather to seasonal scales. The fidelity of the MJO is therefore critical to the fidelity of many modes of variability. The fields shown in the left panel of Fig. 5 show the zonal anomaly of the low pass filtered (20-60 day band) outgoing longwave radiation (OLR) at the top of the atmosphere,as simulated by the nature run. The OLR is an indicator of the temperature of the tallest clouds at any location, such that a large negative anomaly indicates tall (and so cold) clouds and the presence of the deep atmospheric convection associated with the active phase of the MJO. The top-to-bottom progression of fields in the left panel represents the progression, in 5-day increments, of a full cycle of a sample MJO event from the nature run. This event, and the others simulated in the nature run, fall well within the expected MJO behavior from a coupled model simulation^101^.

Many coupled models simulate a credible MJO variability, but an issue common to most General Circulation Models (GCMs) used for weather and climate is the failure of the MJO to propagate from the Indian Ocean to the Western Pacific across the Maritime Continent^102^. The sequence of figures on the right panel of figure 5 show an example of an MJO event that clearly traverses the Maritime Continent. The relatively small number of MJO events that can be represented in the nature run (a 30-60 day oscillation in a year-long simulation) precludes a full statistical evaluation, but the number of MJO events that cross the Maritime Continent in the nature run approximates the ratio of those kinds of events found in observational estimates^103^. This ability to overcome the barrier effect of the Maritime Continent in this high resolution simulation is the subject of ongoing studies.

**Fig. 6:**
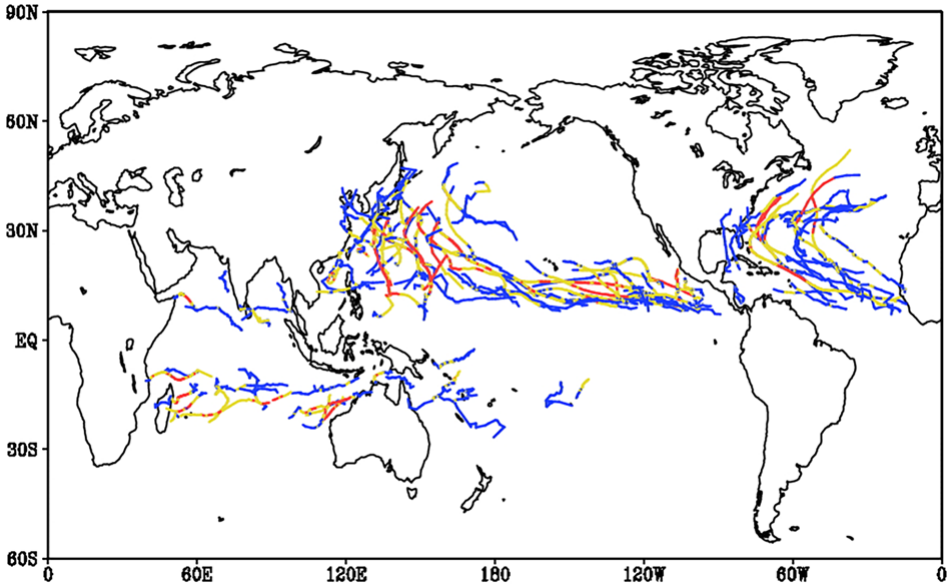
Tracks of the tropical cyclone events simulated in the GEOS/ECCO Nature Run. Tropical storms are shown in blue, hurricanes in yellow and major hurricanes in red.

**Fig. 7:**
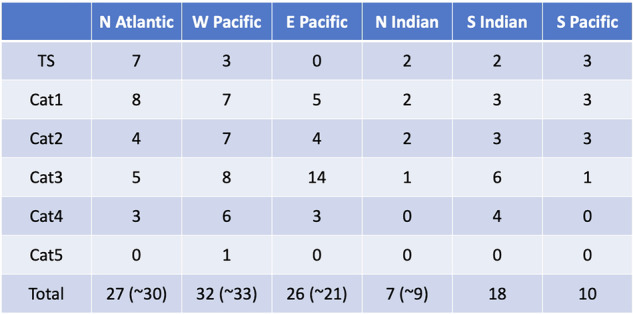
Storm counts of tropical cyclones of different strengths in the different ocean basins as simulated by the GEOS/ECCO Nature Run. TS are tropical storms, Cat1-5 are Hurricane category storms based on the Saffir-Simpson scale, as in^110^, and numbers in parentheses are the total counts based on NOAA’s best track observations as in^110^.

Another important source of atmospheric variability is the occurrence of extreme conditions associated with tropical cyclones. Studies evaluating the frequency and strength of the simulation of tropical cyclones in GCMs generally report that even at resolutions up to 25km the strength and frequency are underestimated^104^. Typical hurricane prediction models are run at resolutions of 3km, termed ‘cloud resolving’, since the processes that contribute to the formation of these clouds are resolved by the grid scale motions, and these models are generally limited in spatial extent. The ability of a nature run to represent tropical cyclones with reasonable fidelity is an important element of its role in OSSE studies, as it enables assessment of the impact of a proposed observing system on the prediction of these potentially destructive extreme events. A depiction of the tropical cyclone events simulated by the nature run is shown in Fig. 6, color-coded to differentiate events of different strength. The table in Fig. 7 reflects the frequency of tropical cyclones and hurricanes of different strengths, and the comparison with observations. The total storm counts, as well as the proportion of storms in the highest three categories are well represented in the nature run.

### Ocean

For the ocean we present time-mean surface currents as compared to ECCO, mixed layer depth as compared to an Argo-based estimate^105^, and temperature, and salinity fields as compared to the time-mean World Ocean Atlas 2023 (WOA23) climatology^106,107^. We also present a comparison of the simulated barotropic tides to the TPXO10 global tidal solutions^108^. Additional evaluation of the tides in the GEOS/ECCO simulation was performed^53^, where it was found that the large-scale patterns of K1 and O1 diurnal internal tides are well reproduced, but it was also found that the barotropic and internal tide structures show some irregular amplitudes, likely due to a misadjustment of internal tide generation and dissipation mechanisms in the model^54^.

#### Mixed Layer Depth and Surface Currents

**Fig. 8:**
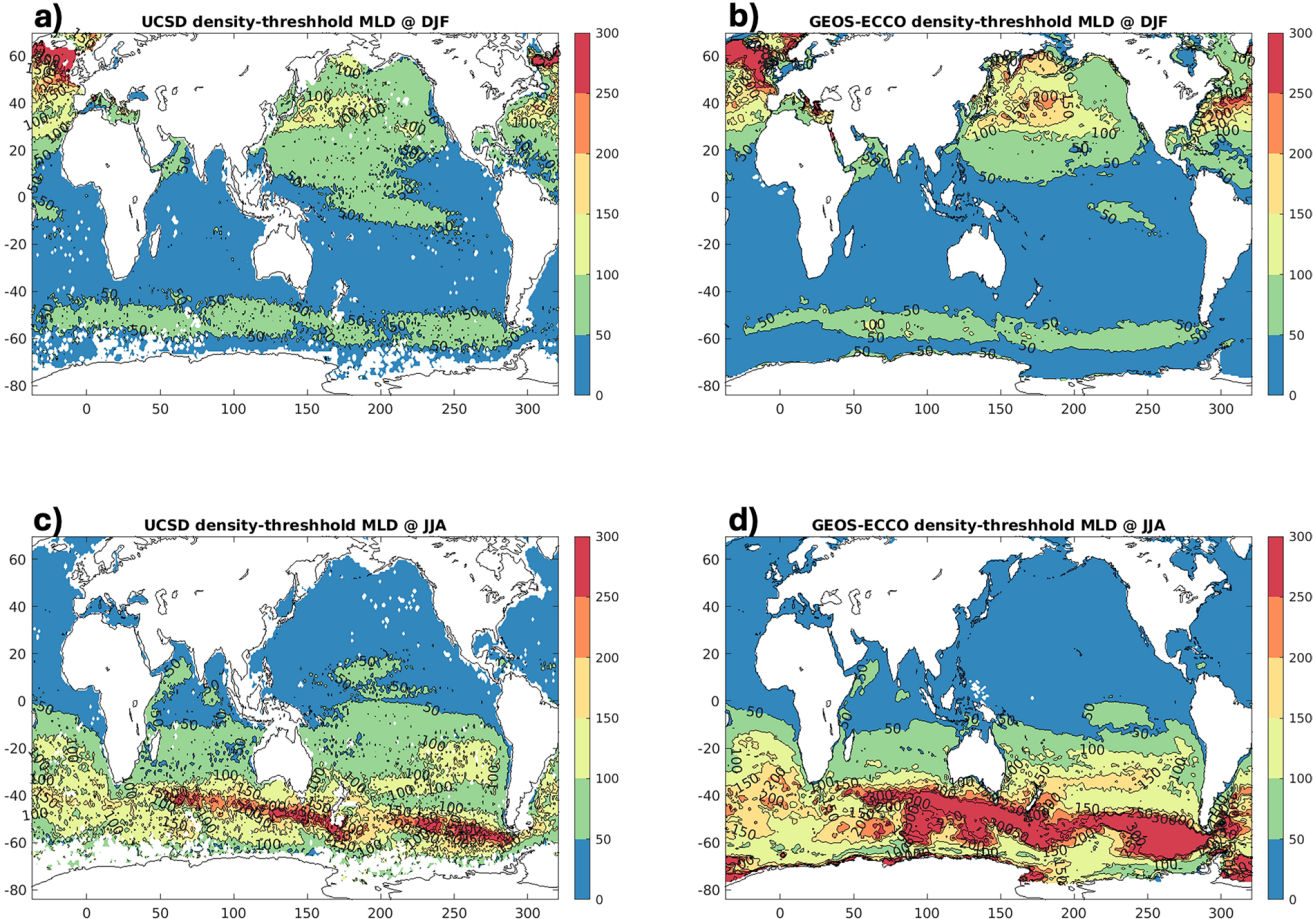
(**a**) December-January-February averaged ARGO-based Ocean Mixed Layer Depth in *m*. (**b**) Same as (**a**) but from the GEOS/ECCO simulation. (**c**) Same as (**a**) but for June-July-August averaged mixed layer depth. (**d**) Same as (**c**) but from the GEOS/ECCO simulation.

Figure 8 shows the seasonal mean ocean mixed-layer depth (MLD) in DJF and JJA. During the boreal wintertime (DJF), the MLD is deepened in Northwest Pacific (subtropical gyre) and Atlantic (subpolar) regions, reaching above 100-m due to convective mixing from surface cooling induced by latent heat exchange, and wind-induced mixing (Figs. 8a,b). During the austral wintertime (JJA), the MLD is deepened in the Southern Ocean (SO) region, reaching above 250-m in the regions with an intense Antarctic Circumpolar Current (ACC) where surface heat loss is enhanced. Mixing in this region is increased due to the interactions of strong surface currents and significant seafloor topography with standing meanders and ocean eddies (Figs. 8c,d). Seasonal MLD maps from GEOS/ECCO simulation are similar to those from the Argo-based estimate^105^ as shown in right versus left panels in Fig. 8. For currents in the SO region, including eddies at the tip of Africa (Agulhas ring), the wintertime MLD depth is larger in GEOS/ECCO compared to that from Argo, more likely due to strong barotropic tidal currents and wind-frontal interactions in MITgcm simulation as discussed in previous studies^53,54^.

**Fig. 9:**
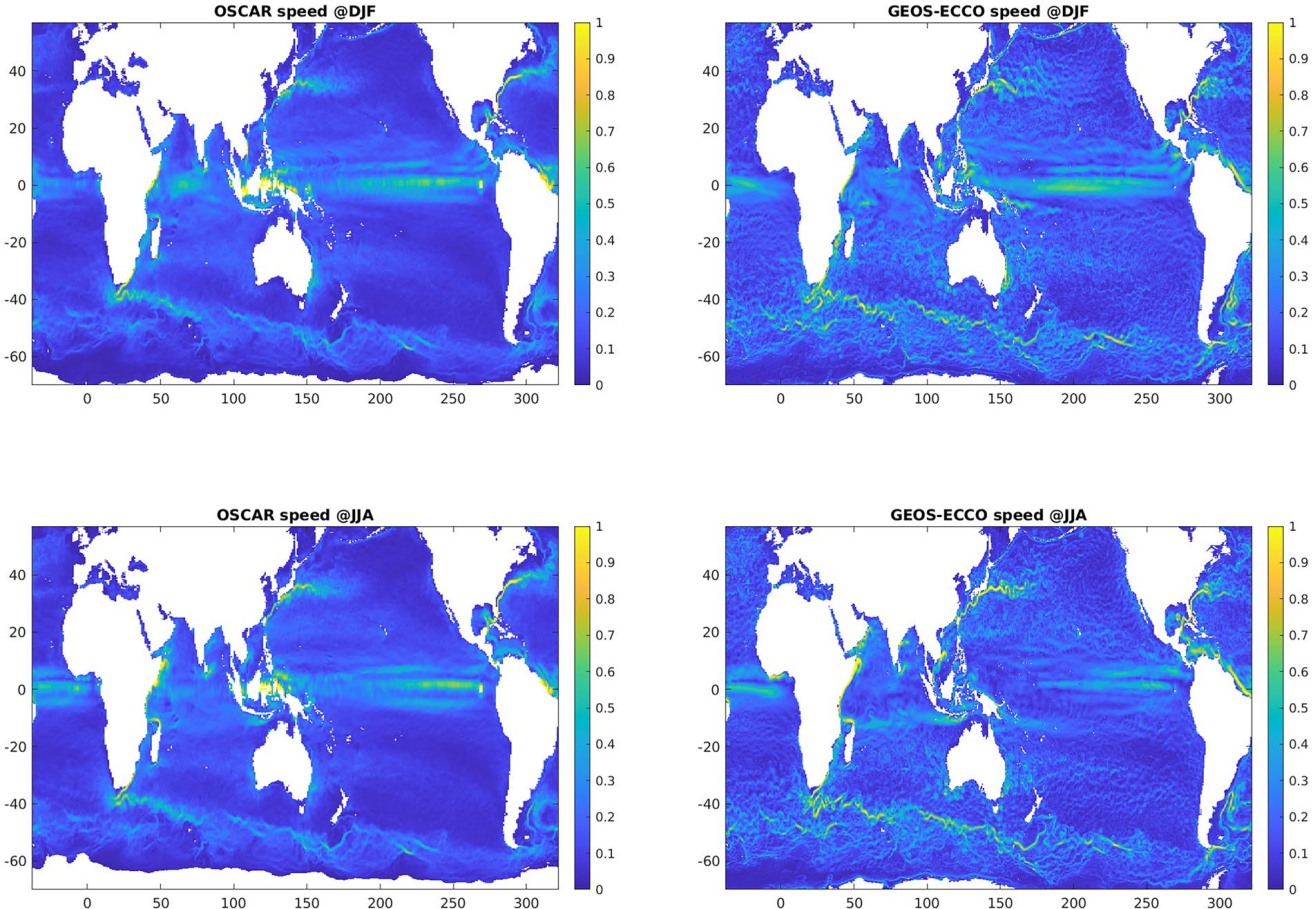
(a) December-January-February averaged Surface Current from ECCO in *m* *s*^−1^. (**b**) Same as (**a**) but from the GEOS/ECCO simulation. (**c**) Same as (**a**) but for June-July-August averaged surface current. (**d**) Same as (**c**) but from the GEOS/ECCO simulation.

Surface currents from the GEOS/ECCO simulation and from observation based estimates of surface currents are obtained from a 2012-2021 mean of the Ocean Surface Current Analyses Real-time (OSCAR)^109^ data are shown in Fig. 9. Surface current magnitudes are larger in the Western Boundary Current regions (e.g. Gulf stream and Kuroshio current) during the boreal wintertime (DJF), and the SO region shows stronger currents during the austral wintertime (JJA). The surface currents in GEOS/ECCO simulation (right hand side panels of Fig. 9) show finer structures and stronger eddy filaments compared to those from OSCAR (left hand side panels of Fig. 9) because the GEOS/ECCO simulation was run at higher horizontal resolution. Interestingly, surface currents in the tropical region in GEOS/ECCO model are weaker in JJA versus those in DJF, more likely due to a slower trade wind during boreal summer (JJA).

#### Time-Mean Temperature and Salinity Fields

**Fig. 10:**
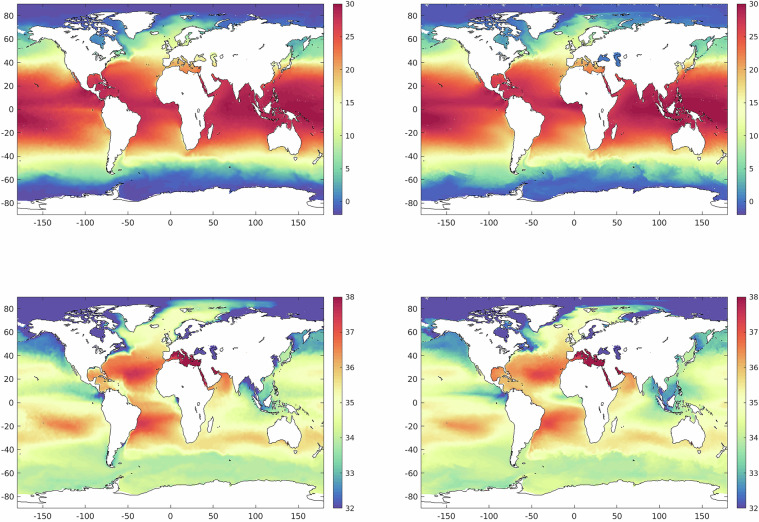
(**a**) Annual mean sea surface temperature in ^°^*C* from WOA23 climatology, (**b**) annual mean sea surface temperature in ^°^*K* from the GEOS/ECCO Nature run, (**c**) annual mean sea surface salinity in PSU from WOA23 climatology and (**d**) annual mean sea surface salinity in PSU from the GEOS/ECCO Nature Run.

**Fig. 11:**
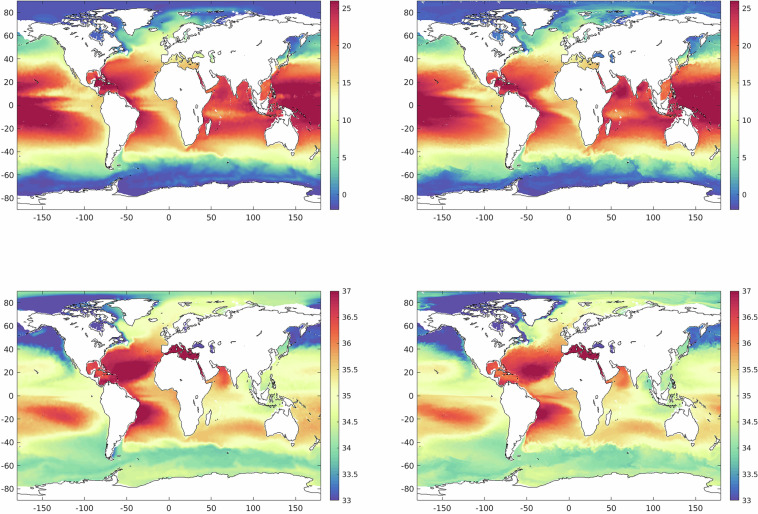
Same as Figure 10 but at 100-m depth into the ocean.

**Fig. 12:**
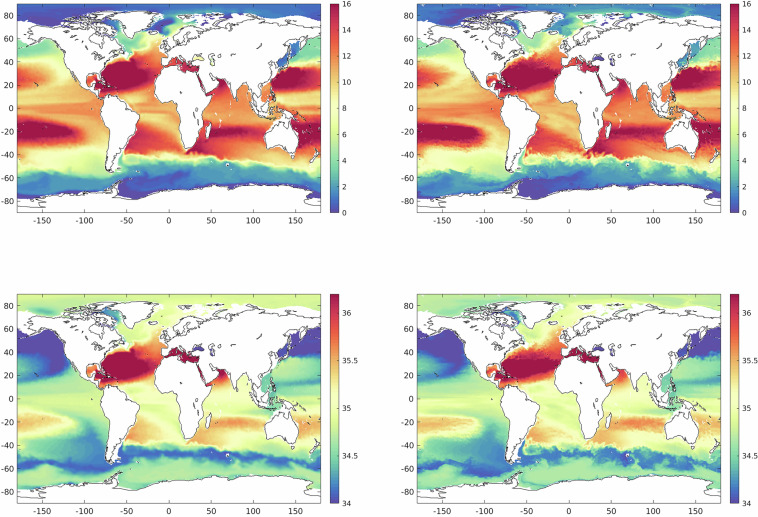
Same as Figure 10 but at 300-m depth into the ocean.

Panels 10a and 10b show a comparison of the annual mean sea surface temperature (SST) fields from WOA23 and the GEOS/ECCO simulation, respectively. The similarity of pattern and magnitude is apparent. The WOA23 SST is warmer in the mid-Atlantic basin by up to 1^*o*^ K and colder than the GEOS/ECCO simulation by a similar amount off the coast of Greenland. The high resolution of the GEOS/ECCO simulation is also apparent in the eddy-rich regions of the Gulf and Kuroshio current regions and in the Atlantic Circumpolar Current. Panels 10c and 10d show a comparable comparison of the sea surface salinity (SSS) fields from WOA23 and the GEOS/ECCO simulation. The largest differences are in the mid-Atlantic region, where the GEOS/ECCO simulation is fresher than WOA23 estimates. The panels in Fig. 11 show an analagous set of comparisons as Fig. 10, except at 100m depth into the ocean, which is included here to demonstrate the fidelity of the simulation in the upper ocean below the mixed layer. Temperature at 100-m depth is significantly cooler compared to the SST in the tropical region (in particular, over the East Pacific and East Atlantic oceans) because 100-m is the edge of thermocline marking the bottom of the upper surface layer. Consistently, the salinity at subsurface layer 100-m is slightly saltier than the SSS, an indication of the fresher water near the surface probably due to competition of condensation and evaporation at the ITCZ. Temperature/salinity structures at 100-m depth show similar structure in the GEOS/ECCO nature run and the WOA23 (Fig. 11) fields. The panels in Fig. 12 show an analagous set of comparisons as Fig. 10, except at 300-m depth into the ocean within the pycnocline layer. At this layer, the temperature is cooler and saltier compared to the ocean surface and sub-surface (Figs. 10, 11, and 12). The differences between the GEOS/ECCO nature run and the WOA23 annual mean fields are comparable in pattern and magnitude to the differences shown at the surface.

**Fig. 13:**
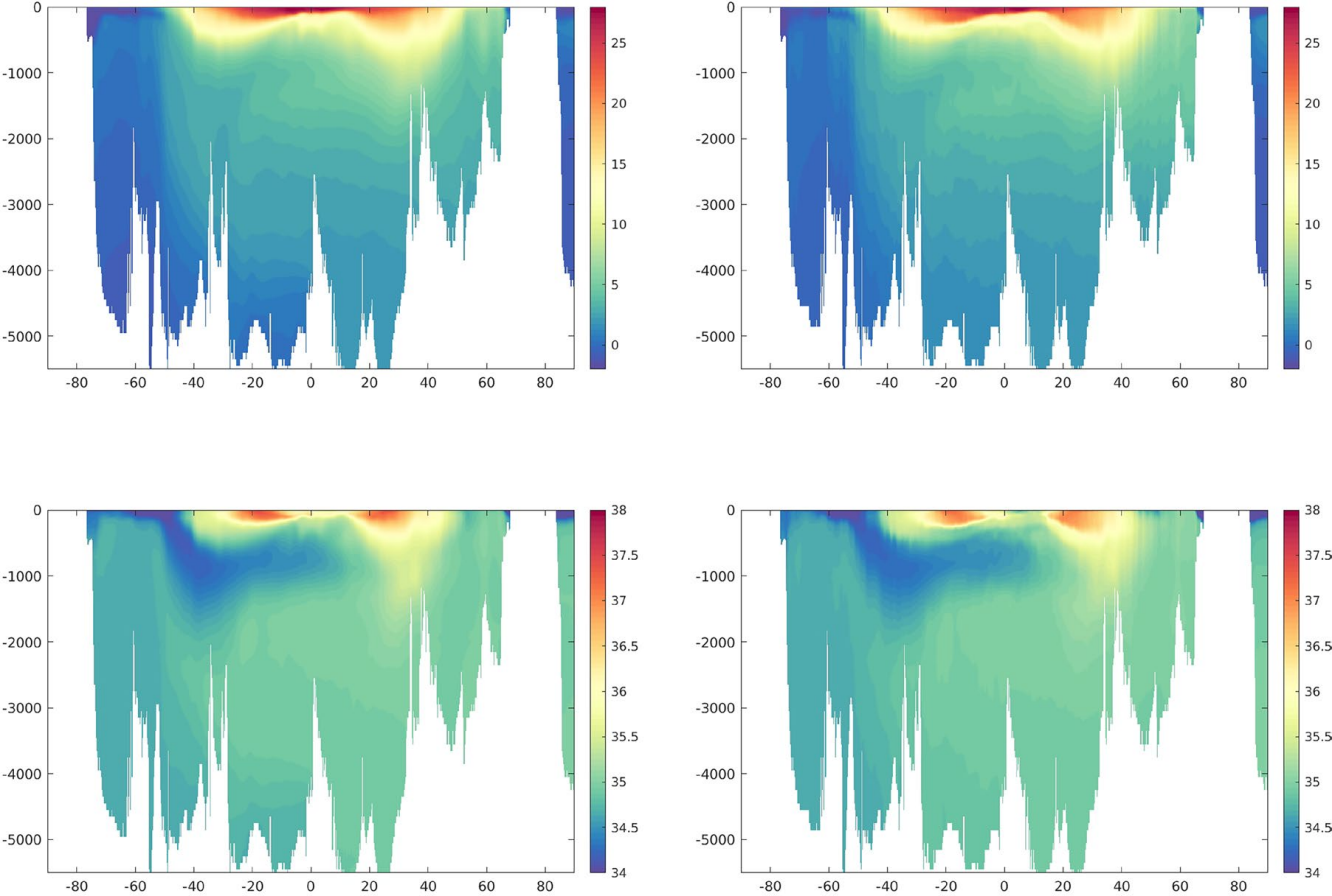
(**a**) Annual mean potential temperature as a function of depth and latitude at 30^°^W longitude in ^°^*C* from WOA23 climatology, (**b**) annual mean potential temperature as a function of depth and latitude at 30^*o*^W longitude in ^°^*C* from the GEOS/ECCO Nature run, (**c**) annual mean salinity as a function of depth and latitude at 30^°^W longitude in PSU from WOA23 climatology and (**d**) annual mean salinity as a function of depth and latitude at 30^°^W longitude in PSU from the GEOS/ECCO Nature Run. The insert illustrates the location of the cross section with a red line.

**Fig. 14:**
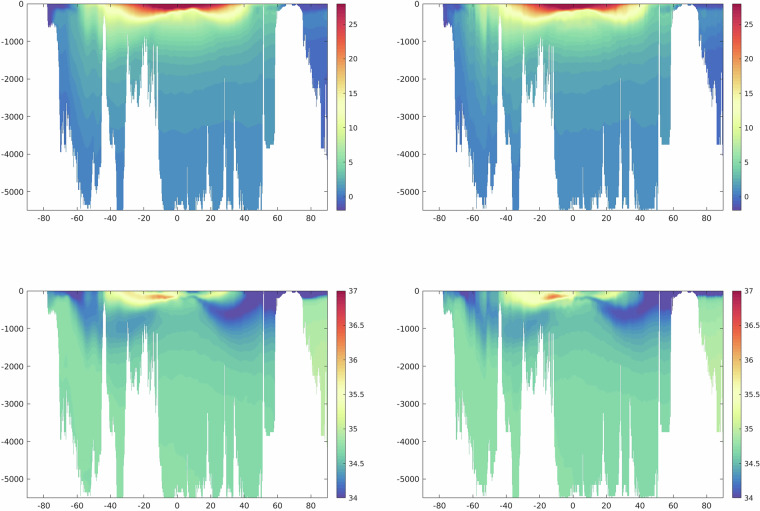
Same as Figure 13 but along the 180^°^E meridional section.

**Fig. 15:**
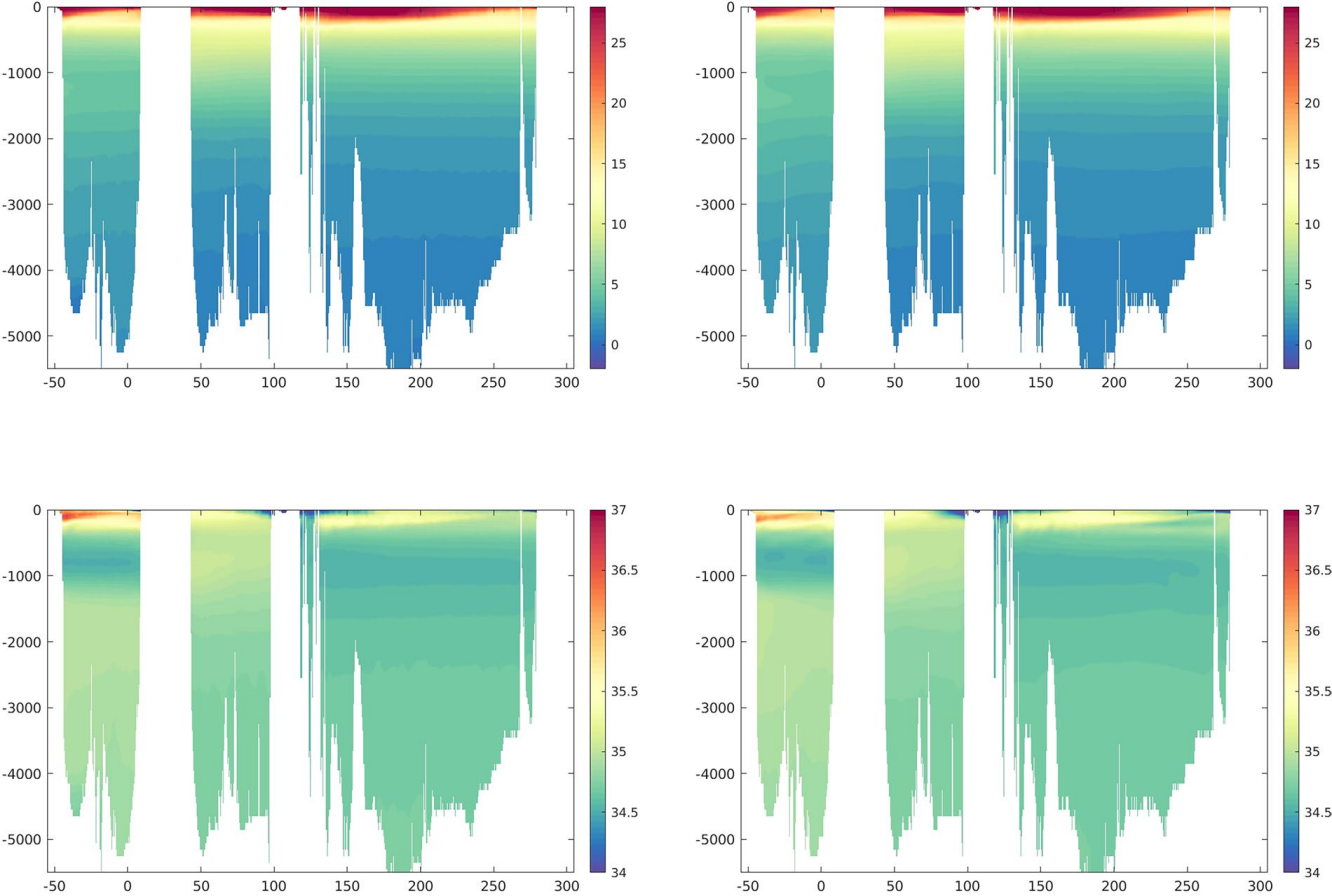
Same as Figure 13 but along the Equator as a function of Longitude.

The fidelity of the simulated ocean below 300m depth in the GEOS/ECCO nature run is demonstrated by evaluating the potential temperature and salinity as a function of depth at two latitudinal “cut”s, one in the Atlantic and one in the Pacific, and at one longitudinal “cut” at the equator. Figure 13 shows the longitudinal cut at 30^°^*W* in comparison with WOA23. Fig. 13a and b show that the modeled and WOA23 temperature cross-sections are almost indistinguishable in structure and magnitude. The cooler temperatures in the nature run that were seen at the surface and at 300m depth are apparent here, along with a warmer model temperature at depths below 3km in the tropics, shown as a weaker cooling with depth than seen in WOA23. Figure 14 shows, similar to the Atlantic cross section, that the potential temperature and salinity as a function of depth in the Pacific basin of the nature run are almost indistinguishable from WOA23 climatological fields. Figure 15 shows that across the equator the behavior of the modeled and WOA23 temperature and salinity are also almost indistinguishable.

#### Evaluation of Tides

**Fig. 16:**
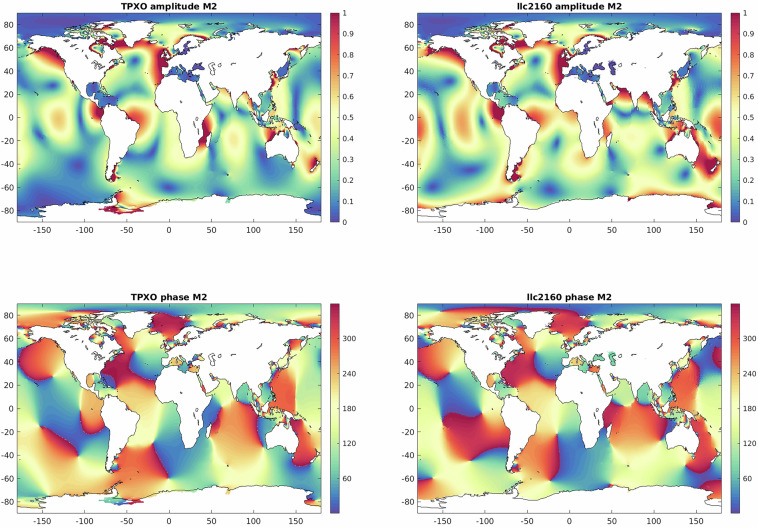
(**a**) M2 tidal amplitude in m from the TPXO10 global tidal solution, (**b**) M2 tidal amplitude in m from the GEOS/ECCO nature run, (**c**) M2 tidal phase in degrees from the TPXO10 global tidal solution, and (**d**) M2 tidal phase in degrees from the GEOS/ECCO Nature Run.

One unique feature of the GEOS/ECCO nature run is the simultaneous inclusion of atmospheric and ocean tides. An evaluation of the M2 tidal component is shown in Figure 16. The M2 tidal component is the dominant lunar semi-diurnal tidal component, with a period of about 12 hours, and is the largest and most significant component of tides in most locations. A comparison of Figures 16a and 16b shows a general correspondence of structure between the TPXO and the GEOS/ECCO nature run tidal amplitude, with the nature run showing higher amplitudes in the Pacific and Indian Ocean. The M2 tidal phase comparison, shown in Figs. 16c and 16d, show a general correspondence of phase in the western Atlantic and Pacific basins, but up to 20 degrees out of phase in the southeastern Pacific, north western Pacific and western Indian Ocean regions.

## Usage Notes

The GEOS output fields that include atmospheric prognostic fields, atmospheric diagnostic fields related to the hydrological cycle and radiation, and fluxes at the air-surface interface, all appear in COARDS-compliant netcdf format. The output horizontal grid is the GEOS native cubed sphere grid, and the latitude and longitude of each grid cell are available in an external netcdf file that appears with the model output on the public-facing disk referred to in the Data Records section. The output vertical grid is a pressure coordinate, and fields were interpolated from the model’s native hybrid eta-pressure coordinate^56^ to uniform pressure levels.

The MITgcm output that includes ocean prognostic and diagnostic fields appears in binary files. The output horizontal grid is the native “lat-lon-cap” grid, and the fields that describe the grid, such as the grid centers, corners and angles, are found in: grid. The depth locations of the vertical grid are also found in the grid directory.

## Data Availability

The dataset described here is available at https://www.poseidon-ocean.net/products/datasets/^93^, and instructions for downloading the data can be found at: https://www.poseidon-ocean.net/access-process-for-the-dyamond-dataset-on-sciserver/.

## Code availability

The code that was used to generate the GEOS/ECCO nature run consists of components from GEOS, MITgcm, and a geos/mitgcm coupling layer. All of this code is available to the public at: code. In order to conduct the GEOS/ECCO nature run simulation, additional external files are needed, containing information such as aerosol emissions, land surface vegetation cover and greenness, atmospheric gas constituent concentrations, and land mask information. These fields are available upon request from the authors.
